# MicroRNA networks regulate the differentiation, expansion and suppression function of myeloid-derived suppressor cells in tumor microenvironment

**DOI:** 10.7150/jca.35205

**Published:** 2019-07-10

**Authors:** Yanping Su, Ye Qiu, Zhidong Qiu, Peng Qu

**Affiliations:** 1 Department of Histology and embryology, Shangdong First Medical University & Shangdong Academy of Medical Sciences, Taian, Shangdong, China.; 2 National Engineering Lab for Druggable gene and protein screening, Northeast Normal University, Changchun, Jilin, China.; 3 Department of pharmacy, Changchun University of Chinese Medicine, Changchun, Jilin, China.; 4 National Cancer Institute, National Institutes of Health, Frederick, MD, USA.

**Keywords:** MicroRNA, Myeloid-derived suppressor cells, Tumor

## Abstract

Myeloid-derived suppressor cells (MDSCs), one heterogeneous population of immature myeloid cells, have suppressive function on immune response during tumor, inflammation, infection and autoimmune diseases. The molecular mechanism underlying expansion and function of MDSCs is becoming appreciated to manipulate immune response in the diseases. MicroRNA (miRNAs) as one short noncoding RNAs, are involved in regulating cell proliferation, differentiation and maturation. However, it needs to be further studied how miRNAs mediate the development and function of MDSC in association with cancer and other diseases. In the review, we report and discuss recent studies that miRNAs networks regulate the differentiation, expansion and suppression function of MDSCs in tumor microenvironment or other diseases through different signaling pathways. Those studies may provide one novel potential approach for tumor immunotherapy.

## Introduction

Myeloid-derived suppressor cells (MDSCs) are the progenitors of myeloid cells with suppressive function on immune response in tumor microenvironment (TME). In tumor bearing-mice, MDSCs are generally characterized as GR-1^+^CD11b^+^ cells, which are further divided as two subtypes: CD11b^+^Ly6G^-^Ly6C^high^ monocytic MDSCs and CD11b^+^Ly6G^+^ Ly6C^low^ granulocytic MDSCs 1, 2. They utilize different suppressive mechanisms to inhibit the antitumor immune response. Monocytic MDSCs regulates immune suppression through the production of NO and arginase^3^. In contrast, the inhibition of granulocytic MDSCs is regulated via ROS and H_2_O_2_ 4. In patients with cancer, there are different types of MDSCs. In general, MDSCs are characterized as CD33^+^CD15^+^CD14^-^HLA-DR^low^ populations 5. Many factors, such as cytokines, growth factors and microbial products released in tumor microenvironments have been shown to be involved in the induction and expansion of MDSCs with suppressive activity 6, 7. Most of these mediators activate signaling pathways in tumor MDSCs that involve NF-κB and Signal transducer and activator of transcription (STATs) 1, 8, 9 . Some novel regulatory mechanisms for the differentiation, expansion and suppression of tumor MDSCs were described recently. There is emerging evidence that microRNAs (miRNAs) cooperate transcriptional factors to become complex regulatory networks which mediate tumor MDSCs 10, 11.

miRNAs are the abundant small, single-stranded, non-coding RNA of about 22 nucleotides. miRNAs base-paired with the complementary sequence within target mRNAs to mediate post-transcriptional gene repression or target mRNA degradation 12. The stability of the miRNA-mRNA interaction is critical for repressing the potential target 13. Each mRNA could be targeted by different miRNAs and a single miRNA may target different mRNAs 14, 15. Gene expression silencing by means of miRNAs and changes in the miRNA expression level regulate various biological processes, including the differentiation, maturation, function of immune cells and maintenance of immune homeostasis 16-19. MDSCs , an immune-suppressive cell, plays an important role in a wide range of human diseases including cancer, chronic inflammatory and autoimmune diseases. Therefore, both abnormal expression and function of miRNAs in MDSCs were investigated, so that novel miRNAs regulatory mechanisms on MDSCs were displayed.

## MicroRNAs regulate the differentiation and activation of tumor MDSCs

### 1. miRNAs up-regulation on tumor MDSCs

**Figure 1:**
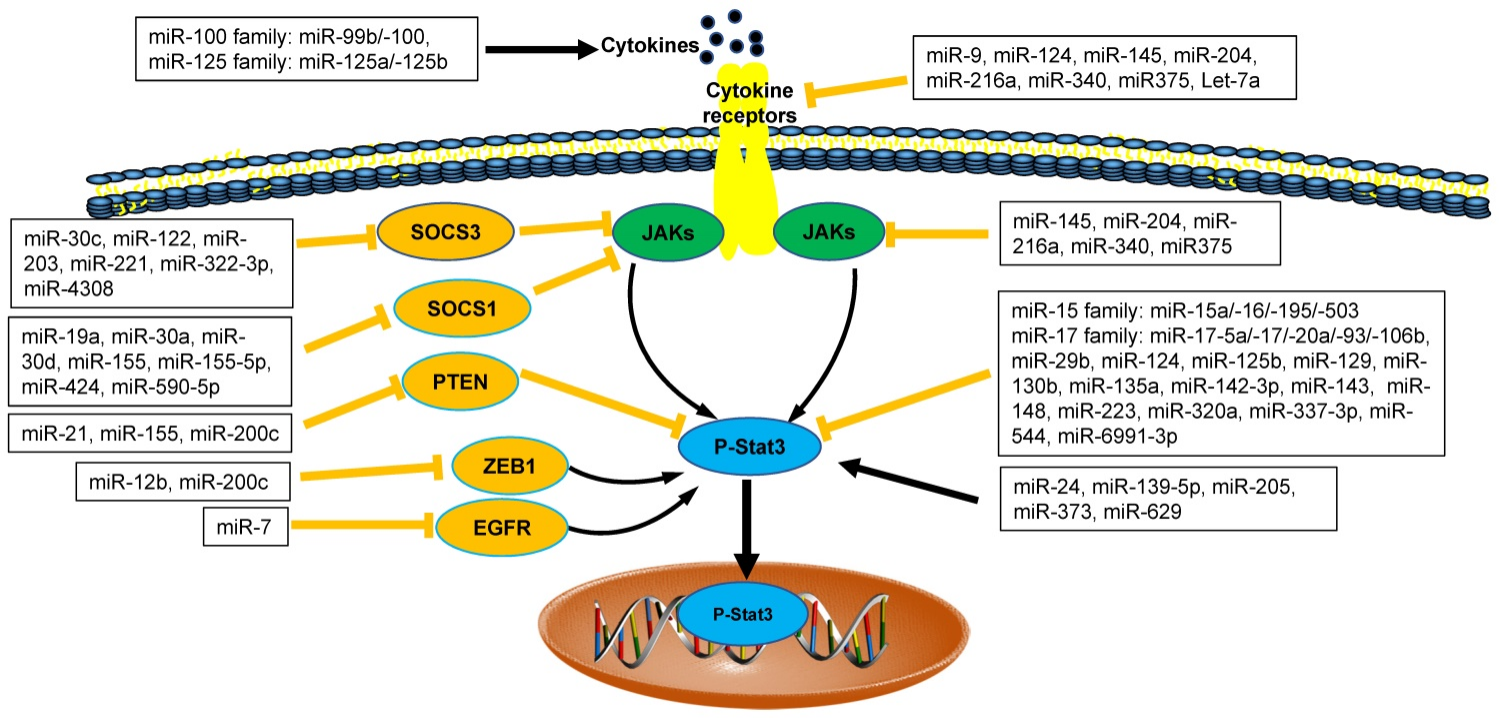
** The interaction between microRNAs and Stat3 in tumor microenvironment.** MicroRNAs (miRNAs) are emerging as direct or indirect regulators of Janus Kinase (JAK)-Signal Transducer and Activator of Transcription 3 (STAT3) pathways in the pathogenesis of cancer. In each process, microRNAs networks play positive (black line) or negative (orange line) roles. SOCS1: Suppressor Of Cytokine Signaling 1; SOCS3: Suppressor Of Cytokine Signaling 3; PTEN: Phosphatase and Tensin Homologue; ZEB1: Zinc Finger E-Box Binding Homeobox 1; EGFR: Epidermal Growth Factor Receptor.

**Figure 2:**
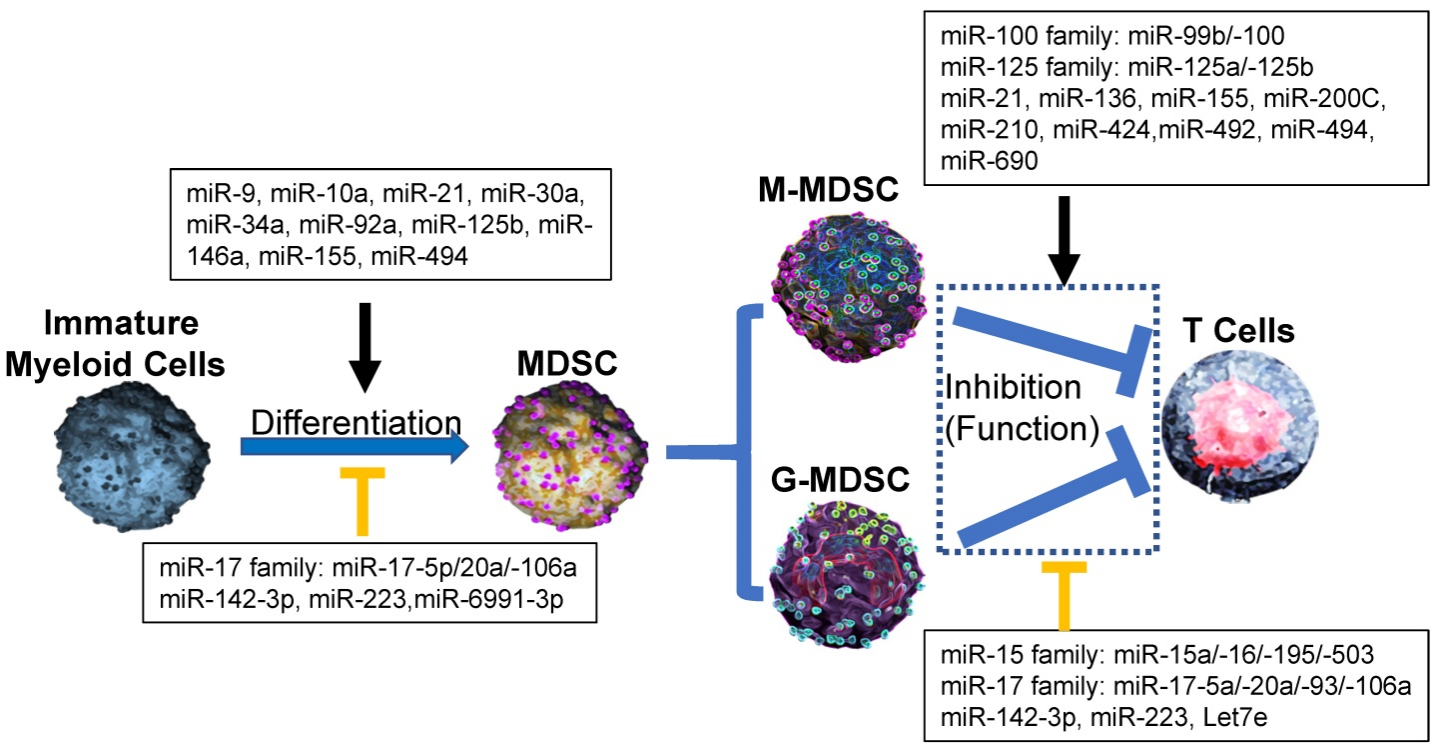
** Effect of microRNA networks on MDSCs' differentiation, expansion, activation and function in tumor microenvironment.** In tumor microenvironment, MDSCs from Immature myeloid cells are divided as two subtypes: monocytic MDSCs (M-MDSCs) and Granulocytic MDSCs (G-MDSCs), which utilize different suppressive mechanism to inhibit the antitumor ability of T cells. In each process, microRNAs networks play positive (black line) or negative (orange line) roles.

Recent reports demonstrated that miR-494 expression in tumor MDSCs was dramatically induced by tumor-derived factors, such as TGF-β1 to regulate the accumulation and activity of MDSCs by targeting of phosphatase and tensin homolog (PTEN) and activation of the Akt pathway 20. miR-10a activated AMPK signaling to promote expansion and activation of MDSCs in breast cancer cells with chemotherapy-induced immune resistance 21. miR-6991-3p could directly target the immune checkpoint gene LGALS9 (Galectin 9) and MiR-6991-3p mimic transfection suppressed expansion and promoted apoptosis of MDSCs through suppressing LGALS9-mediated activation of Janus kinase (JAK) and STAT3 22. In B lymphoma -bearing mice, miR-30a expression was increased in both G-MDSCs and M-MDSCs. After the transfection of miR-30a mimics, the differentiation and suppressive abilities of MDSCs were increased via up-regulation of arginase-1. miR-30a also down-regulated suppressor of cytokine signaling 3 (SOCS3) mRNA to activate STAT3 signaling to further promote MDSC differentiation and suppressive activities, indicating that same individual microRNA could regulate differentiation and activity of MDSCs through difference pathways 23, 24 (Figure 1). The inhibition of miR-9 promoted the differentiation of MDSCs with significantly reduced immunosuppressive function via by targeting the runt-related transcription factor 1 (Runx1), an essential transcription factor in regulating MDSC differentiation and function 25 (Figure 2).

### 2. Inhibitory roles of miRNAs on tumor MDSCs

miRNAs also negatively mediated the differentiation and activity of tumor MDSCs. The overexpression of miR-17 family members such as miR-17-5p, miR-20a and miR-106a in human progenitor cells repressed AML1 by binding to its promoter, which resulted in the down-regulation of M-CSFR, thus limiting MDSC differentiation 26 (Figure 2). In LLC and ovarian carcinoma models, miR-223 suppressed differentiation and accumulation of MDSCs by targeting molecule myocyte enhancer factor 2C (MEF2C) 27. miR-142-3p could prevent MDSC differentiation during tumor-induced myelopoiesis by modulating STAT3 and C/EBPβ signal pathway, indicating that the potential therapeutic application for miR-142-3p oligonucleotide as adjuvant tool for adoptive T cell therapy of cancer 28 (Figure 1).

### 3. miRNAs from tumor-derived extracellular vesicles

Extracellular vesicles (EVs) were involved in miRNAs regulation on MDSCs. Cancer cells secreted EVs, which were involved in the intercellular transfer of proteins, lipids, and genetic material (such as miRNAs). Those tumor-associated EVs represented an ideal candidate due to their ability to recirculate in body fluids during the process of MDSC generation from bone marrow in tumor microenvironment 29-31. In melanoma patients, some miRNAs (such as miR-99b, miR-100, miR-125a/-125b, miR-146a/-146b, miR-155, let-7e), which were highly detected in plasma as associated with EVs, mediated the generation and functional features of tumor M-MDSCs 8, 32, 33. In Acute myeloid leukemia (AML), miR-34a promoted the expansion of MDSCs as the regulatory mechanism by which Mucin 1, cell surface associated (MUC1) drives c-myc expression in Acute AML cells and tumor-derived EVs 30. In addition, miR-34a also inhibited the apoptosis of MDSCs via targeting N-myc 34 or p2rx7/Tia1 35 (Figure 2). Those data suggested that miR-34a upregulated the generation and accumulation of tumor MDSC through different pathways as many other miRNAs.

### 4. miRNAs from tumor-derived exosomes

Exosomes derived from tumor (such as gliomas) were also involved in MDSC differentiation. In glioma-bearing mice, glioma-derived exosomes (GDEs) facilitated the expansion and function of MDSCs. Hypoxia promoted the upregulation of miR-10a and miR-21 expression in GDEs to induce MDSC activation by targeting the IκBα/NF-κB and PTEN/PI3K/AKT pathways. The reduced numbers of MDSCs were observed in the spleens of mice bearing miR-10a or miR-21 knockout glioma cells, compared with those in bearing glioma cells 36. Those GDEs also regulated the expansion of MDSCs through miRNA-29a/Hbp1 and miRNA-92a/Prkar1a pathways 37, indicating that GDEs could regulate MDSC expansion through difference miRNAs (Table 1).

## miRNAs mediate the function of MDSCs in tumor microenvironment

### 1. miRNAs regulate MDSCs through Stat3 pathway

We ever discussed the findings about the relationship between miRNAs and JAK/STAT3 in cancer 2, 8. Recently, there were more emerging data about negative and positive regulation of miRNAs networks and JAK/STAT3 signaling pathways via direct and/or indirect regulatory mechanisms in tumor microenvironment (Figure 1). STAT3, as an important transcript factor, is also required for the suppressive function of tumor MDSCs 2, 38-40. Therefore, we focused on the regulation of miRNAs on tumor MDSC through JAK/STAT3 further.

Recent data demonstrated that four members of miR-17 family (including miR-17, miR-20a, miR-93, miR-106a) played the inhibitory roles in the function of tumor MDSCs. In tumor microenvironment, tumor-associated factors downregulated the expression of miR-17-5p and miR-20a and promoted the Stat3-associated suppressive function of MDSCs 41. Thus, miR-17-5p and miR-20a may potentially be used as targets in immunotherapy strategies to inhibit the function of MDSCs via reducing STAT3 expression 42.

The enhanced expression of miR-142-3p reduced the immunosuppressive activity of tumor BM-MDSCs, restoring CD8+ T cell proliferation through inhibiting C/EBPβ/STAT3 pathway 28. miR223 and Let7e also downregulated the suppressive function of MDSCs through inhibiting the activation of STAT3 in Gliomas 41. The expression of PD-L1 on tumor MDSCs, which was closely related to the suppressive function of MDSCs was regulated by the miR-93/106b miRNA cluster of miR-17 family through stat3 pathway. Those PD-L1 expression levels on MDSCs could be reduced significantly after treatment of miR-93 mimics 43, 44. Therefore, those miRNAs above regulated tumor MDSCs plasticity through inhibiting STAT3 pathways (Figure 1).

The regulatory roles of miRNAs on tumor MDSCs are positively involved in STAT3 pathway. miR-200c promoted suppressive potential of tumor MDSCs by targeting PTEN/friend of Gata 2 (FOG2), which led to STAT3 and PI3K/Akt activation 45. miR-155 and miR-21 demonstrated a synergistic effect on MDSC induction via targeting SHIP-1 and PTEN respectively, leading to Stat3 activation 46. In a line with this finding above, MDSCs were shown to require miR-155 to facilitate tumor growth 47. However, recent study revealed the loss of miR-155 in MDSCs enhanced its recruitment and function in solid tumor, which was not consistent with the results above 11, 48.

### 2. miRNAs regulate MDSCs through PD-L1/PD-1 pathway

The immunotherapy of checkpoints PD-L1/PD-1 on tumor have been broadly applied and those checkpoints were also associated with the tumor MDSCs 1, 8. But the interaction between miRNAs and checkpoints PD-L1/PD-1 on tumor MDSCs was recently investigated further. Some scientists reported that five members of miR-15 family, which included miR-15a, miR-15b, miR-16, miR-195 and miR-503, activated T cell response by inhibiting the function of MDSCs and/or Tregs in the tumor microenvironment through blocking PD-L1/PD-1 signaling pathway 42, 45, 49, 50, however, miR-424(322), another member of miR-15 family was inversely correlated with PD-L1 pathways. The high level of miR-424 (322) in the tumor was positively correlated with the function of MDSCs and Tregs 51 (Figure 2). The latest results demonstrated that hypoxic tumor-derived exosomes (TEXs) enhanced the suppressive roles of MDSCs on γδ T cells through a miR-21/PTEN/PD-L1 pathway in oral squamous cell carcinoma (OSCC) 52. Thus, interaction between miRNAs network and PD-L1/PD-1 regulated the expansion and function of tumor MDSCs, providing one novel therapy method for inhibiting MDSC-associated tumor metastasis (Table 2).

### 3. miRNAs regulate MDSCs through other molecular pathways

miRNAs networks regulate the function of MDSCs through other target genes. Recent studies demonstrated that EL-4 tumor-elicited MDSCs showed increased expression of miR-690 with attenuated C/EBPα expression 50. Hypoxia-induced miR-210 enhanced the immunosuppressive activity of tumor MDSCs by increasing arginase activity, nitric oxide production and IL-16 53. It was reported that the expansion of tumor MDSCs was regulated by miR-494 through PTEN/AKT. The downregulation of PTEN by miR-494 enhanced the activity of AKT to promote the accumulation of MDSCs in tumor tissues 20. miR-492 also played the similar roles in the suppressive function of MDSCs 54. In melanoma patients, several miRNAs (including miR-100 family member-miR-99b/-100 and miR-125 family members-miR-125a/-125b) induced the activity and accumulation of MDSCs through IL-6 and CCL2, activating JAK/STAT3 pathway further 32 (Figure 1).

## MicroRNAs regulate the expansion and function of MDSCs in inflammation, infection and autoimmune diseases

### 1. Inflammation and infection

MDSCs also play an important role in other pathological conditions, including inflammation, infection and autoimmune diseases 2, 10, 55. Thus, we examined if miRNAs had regulatory roles on MDSCs in those diseases. In mouse model with chronic asthma, miR-20b Induced the increased numbers of MDSCs in lung through TGF-β to inhibit airway inflammation^56^. MDSCs enhanced late sepsis development through immunosuppressive function in mice. miR-375 also regulated the function and miR-21 expression of those MDSCs through targeting JAK2 and further impairing STAT3 in the mice with sepsis 57. miR-21 and miR-181b coupled with NFI-A to promote immunosuppression of MDSCs for improving late-sepsis survival 58. The overexpression of some miRNAs was induced by the synergistic effect of STAT3 and C/EBPβ, which activated miR-21 and miR-181b promoters after sepsis initiation 59. The latest results demonstrated that S100A9 stabilized those STAT3/C/EBPβ protein complex to promote MDSC expansion and immunosuppression in late/chronic sepsis by inducing the expression of miR-21 and miR-181b 60. In inflammation environment, TNFα-mediated miR-136 also targeted NFI-A to induce differentiation and activity of MDSCs 61. MDSCs and Tregs were developed during chronic hepatitis C virus (HCV) infection. miR-124 downregulated the expression level of STAT3, as well as TGF-β, which were overexpressed in MDSCs to reduce the frequencies of MDSCs and Tregs, thus uncovering a novel mechanism for the expansion of MDSC and Tregs during HCV infection 62.

### 2. Autoimmune Disease

Recent studies demonstrated that MDSCs were involved in autoimmune diseases. In experimental autoimmune encephalomyelitis (EAE), MDSCs can suppress T cell activities, in which miR-223 downregulate the number and function of MDSCs via STAT3. In miR-223 knockout mice, MO-MDSCs suppressed T cell proliferation in vitro and EAE in vivo more than wild-type MO-MDSCs^63^ (Table 3).

## Concluding remarks

MDSCs are one of important immune suppressive cells in tumor microenvironment and may be next breakthrough target for tumor immunotherapy 5, 6, 64. The expansion and function of tumor MDSCs have been widely investigated, however, the regulatory mechanism of MDSCs need be further defined 5, 55, 65. Recently, the novel research field of miRNA regulation on tumor MDSCs plasticity were opened 32, 66, 67. The differentiation and function of MDSCs seems to be regulated by multiple miRNAs, some of which were classified by us based on their family members, in order that the scientists may investigate the regulatory roles of other related members of miRNAs family on tumor MDSCs. However, it remains to be clarified how those dysregulated miRNAs were combined *in vivo* to act on MDSCs on key signaling pathway. In addition, most of research data about miRNA function on tumor MDSCs were gained from murine studies, even though there are a few miRNA data from human patients with cancer. The significant and application of miRNAs for the expansion and function of MDSCs in patients with cancer need be further investigated. Therefore, the interaction of dysregulated miRNAs on MDSCs with transcription factors, cofactors and chromatin modifiers may target specific miRNA-regulated pathways to provide novel ways to treat MDSCs in tumor microenvironment.

## Figures and Tables

**Table 1:** microRNAs regulation on the differentiation and expansion of tumor MDSCs

MicroRNAs	Target genes	References
miR-9	Runx1	25
miR-10a	AMPK	21
miR-10a/-21	Rora/NF-κB	36
miR-17-5p/-20a (miR-17 family)	AML1	26
miR-106a (miR-17 family)	AML1	26
miR-30a	SOCS3/Stat3	24
miR-34a	MUC-1	30
miR-34a	N-myc	34, 35
miR-92a	Prkar1a	37
miR-125b	TNF	8
miR-146a	CSF-1R	33
miR-142-3p	C/EBPβ/Stat3	28
miR-155	C/EBPβ	46, 47
miR-223	MEF2C	27
miR-494	PTEN	20
miR-6991-3p	Stat3	22

**Table 2:** microRNAs mediate the function of MDSCs in tumor microenvironment

MicroRNAs	Target genes	References
miR-15a (miR-15 family)	PD-1/PD-L1	50
miR-16/195 (miR-15 family)	PD-1/PD-L1	49
miR-503 (miR-15 family)	PD-1/Stat3	42
miR-424(322) (miR-15 family)	PD-1/PD-L1	51
miR-17-5p/-20a (miR-17 family)	Stat3	42
miR-93/-106b (miR-17 family)	Stat3	43, 44
miR-21	Stat3	46
miR-21	PTEN/PD-L1	52
miR-99b/-100 (miR-100 family)	IL-6/CCL2	32
miR-125a/-125b (miR-125 family)	IL-6/CCL2	32
miR-136	NFIA	61
miR-142-3p	C/EBPβ/Stat3	28
miR-155	Stat3	11, 46
miR-155	MCL-1	48
miR-200C	PTEN/FOG2	45
miR-210	NO production	53
miR-223	Stat3	41
miR-492	PTEN	54
miR-494	PTEN	20
miR-690	C/EBPa	50
Let7e	Stat3	41

**Table 3:** microRNAs regulate the expansion and function of MDSCs in inflammation, infection and autoimmune diseases

MicroRNAs	Diseases/ MDSC plasticity	Target genes	References
miR-20b	Asthma/Expansion	TGFβ	56
miR-21	Sepsis/Expansion	NFI-A	58
miR-124	HCV/Suppressive function	Stat3	62
miR-136	Inflammation/ differentiation	NFI-A	61
miR-181b	Sepsis/ Expansion	NFI-A	58
miR-223	EAE/Suppressive function	Stat3	63
miR-375	Sepsis/Expansion	Jak2-stat3	57
